# Single-incision laparoscopic cholecystectomy versus conventional four-port laparoscopic cholecystectomy: a systematic review and meta-analysis

**DOI:** 10.1007/s00464-016-5381-0

**Published:** 2016-12-30

**Authors:** Laura Evers, Nicole Bouvy, Dion Branje, Andrea Peeters

**Affiliations:** 1 Maastricht University Medical Centre, Maastricht University, P.Debyelaan 25, PO Box 5800, 6202 AZ Maastricht, The Netherlands; 2 Department of Surgery, Maastricht University Medical Centre, Maastricht, The Netherlands; 3 Department of Clinical Epidemiology and Medical Technology Assessment, Maastricht University Medical Centre, Maastricht, The Netherlands

**Keywords:** Laparoscopic cholecystectomy, Single-incision, Conventional, Safety, Feasibility

## Abstract

**Background:**

Single-incision laparoscopic cholecystectomy (SILC) might maximize the advantages of laparoscopic cholecystectomy (LC) by reducing postoperative pain and improving cosmesis. However, the safety and feasibility of SILC has not yet been established. This study assesses safety, patient reported outcome measures and feasibility of SILC versus conventional LC.

**Methods:**

Literature search for RCT’s comparing SILC with conventional LC in gallstone-related disease was performed in PubMed and Embase. The conventional LC was defined as two 10-mm and two 5-mm ports. Study selection was done according to predefined criteria. Two reviewers assessed the risk of bias. Pooled outcomes were calculated for adverse events, pain, cosmesis, quality of life and feasibility using fixed-effect and random-effects models.

**Results:**

Nine RCT’s were included with total of 860 patients. No mortality was observed. More mild adverse events (RR 1.55; 95% CI 0.99–2.42) and significantly more serious adverse events (RR 3.00; 95% CI 1.05–8.58) occurred in the SILC group. Postoperative pain (MD -0.46; 95% CI -0.74 to -0.18) and cosmesis (SMD 2.38; 95% CI 1.50–3.26) showed significantly better results for the SILC group, but no differences were observed in quality of life. Operating time (MD 23.12; 95% CI 11.59–34.65) and the need for additional ports (RR 11.43; 95% CI 3.48–37.50) were significantly higher in the SILC group. No difference was observed in conversion to open cholecystectomy or hospital stay longer than 24 h.

**Conclusions:**

SILC does not provide any clear advantages over conventional LC except for less postoperative pain and improved cosmesis. It is questionable whether these advantages outweigh the higher occurrence of adverse events and shortcomings in feasibility. Considering considerable heterogeneity and low methodological quality of the studies it is advisable to perform well-designed RCT’s in the future to address the safety and clinical benefits of SILC.

Cholecystectomy has emerged as the standard surgical treatment for gallstone-related disease [1]. Since laparoscopic cholecystectomy (LC) is associated with reduced post-operative pain, a shorter hospital stay and a quicker convalescence compared to open cholecystectomy, it is accepted as the gold-standard surgical procedure [2]. In conventional LC, a multiport approach is applied, three or four ports are being used, usually four. Traditionally, two ports of 10 mm in size ensure the access of a camera and a clip applier. Manipulation of the gall bladder for adequate exposure of the field of surgery is accomplished with two ports of 5 mm in size [3]. Nowadays, an increasing number of groups use also different sizes of ports, for instance three 5-mm ports and one 10-mm port, using 5-mm clip appliers. Considering the benefits of LC over open laparoscopy, surgeons have attempted to use even less invasive surgical techniques by minimizing the number of incisions or using even smaller ports [4]. Single-incision laparoscopic cholecystectomy (SILC) might be a promising technique by reducing post-operative pain and improving cosmetic result due to the use of one port only [5]. However, there are concerns with regard to safety and feasibility. The SILC technique might impair visualization due to a lack of triangulation, and there is an increased chance of clashing of the instruments [5]. Moreover, fewer instruments can be used within the body at any given time [3, 5]. Therefore, there might be tissue injury to the bile duct or other important structures such as blood vessels supplying the liver [3, 5].

Meta-analyses have already compared SILC with LC, but the evidence in these articles is limited. In some of these articles, the control group did not correspond to traditional four-port LC, but included three-port or miniport LC [4, 6–8]. In this way, the comparison of SILC with LC might be biased due to different techniques used in LC. Moreover, some authors included observational studies because of a lack of published randomized controlled trials (RCTs) on this topic at time of publication, thereby limiting the quality of the evidence [7, 9]. Only one well-conducted meta-analysis by Gurusamy et al. [3] has compared the results of SILC with a proper conventional LC control group. Based on the analysis of nine RCTs, the authors concluded that there was insufficient quality of evidence to determine any clear advantages. In addition, the safety of SILC had yet to be established. However, the SILC procedure has improved and newer devices have been developed, so this review included fairly outdated RCTs. Moreover, they included not only results of SILC, in two RCTs three-port laparoscopy was used as an experimental intervention, which complicates the interpretation of the results. For a sound comparison between SILC and LC, well-designed RCTs with the use of identical/comparable surgical techniques in each trial arm are indispensable. Therefore, the aim of this review is to compare the SILC procedure with a proper traditional four-port LC control group, including recent RCTs.

## Methods

A systematic review with meta-analysis has been conducted to assess safety, patient-reported outcome measures and feasibility of SILC versus conventional LC in patients undergoing cholecystectomy for gallstone-related disease. Conventional four-port LC is defined as two 10-mm and two 5-mm ports. The review was conducted in accordance with the PRISMA Statement for reporting systematic reviews and meta-analyses [10].

### Literature search

Two authors (LE and AP) independently performed a literature search of the databases PubMed and EMBASE. The keywords used were: “Laparoscopic Cholecystectomy”, in combination with “LESS” or “transumbilical” or “SILC” or “SLC” or “single site” or “single incision” or “single acces(s)” or “single port” AND “Laparoscopic Cholecystectomy”, in combination with “LC” or “MLC” or “four port” or “multi port” or “standard” or “conventional”. The search was limited to papers published in English from 1 January 2011 until 24 March 2016.

### Study selection

Two authors (LE and AP) independently reviewed all the titles and abstracts and reached a consensus with referral to a third author (NB) about which papers met the eligibility criteria. Consequently, the full-text papers were retrieved. After checking the papers and crosschecking their reference lists as well, the final selection of studies has been made. Articles were excluded if the control group consisted of three-port or miniport LC, even though the authors stated that conventional LC was performed. Studies that used three 5-mm ports and one 10-mm port were also excluded. Other reason for exclusion was an unclear definition of the control group.

### Data extraction and validity assessment

Two reviewers (LE and DB) extracted the data and assessed the risk of bias. In case of discrepancy, a third reviewer has been approached (AP).

The following information was extracted from each included study: (1) basic study characteristics, (2) patient characteristics, (3) safety, incl. mortality and adverse events, (4) patient-reported outcome measures such as pain score measured post-operative and at days 1, 2 and 7, cosmesis, and quality of life measured 1, 3, 6 and 12 months after surgery, (5) feasibility, incl. conversion to open cholecystectomy, additional ports needed, operating time and hospital stay.

The risk of bias was assessed by using the Cochrane Collaboration’s tool for assessing risk of bias as stated in “The Cochrane handbook for systematic reviews of intervention” [11] and the Cochrane Hepato-Biliary Group module [12]. The following domains were assessed: (1) random sequence generation; (2) allocation concealment; (3) blinding of participants and personnel; (4) blinding of outcome assessment; (5) incomplete outcome data; (6) selective outcome reporting; (7) for-profit bias. A judgement of “low risk” of bias, “high risk” of bias or “unclear risk” was assigned and elucidated with a comment or quote.

### Data analysis

#### Summary measures

For binary outcomes such as mortality, adverse events, conversion to open cholecystectomy and the need of additional ports, a risk ratio (RR) with 95% confidence interval (CI) was computed. For continuous outcomes such as the VAS score, operating time and hospital stay, the mean difference (MD) with 95% CI was calculated. For outcomes in which different scales are used such as quality of life and cosmesis, the standardized mean difference (SMD) with 95% CI was calculated.

#### Synthesis of results

The meta-analysis was conducted by using the computer program RevMan 5.3 [13]. The statistical method for binary outcomes was the Mantel–Haenszel method, given the few events. The statistical method for continuous outcomes was the inverse variance method. Heterogeneity was explored with the *χ* ^2^ test with significance set at a *p* value of 0.10, and the quantity of inconsistency was measured by the *I* ^2^ statistic. The following thresholds for interpretation of *I* ^2^ were maintained: 30–60%—moderate heterogeneity; 50–90%—substantial heterogeneity; 75–100%—considerable heterogeneity.

A fixed-effect model or a random-effects model was used for pooling depending on the found heterogeneity. The models used for the analysis and the results in the form of a Forrest plot are presented in the paper in “Results” section. In few instances, the SD of the continuous outcome measures was not available. In these cases, a SD was calculated from the available mean and range according to the methods of Hozo et al. [14].

#### Publication bias

“The Cochrane handbook for systematic reviews of intervention—10.4.3.1 recommendations on testing for funnel plot asymmetry” [11] advises to explore publication bias with visual asymmetry on a funnel plot when 10 or more trials are identified. As only nine studies were included in this review, the power was too low to distinguish chance from real asymmetry so publication bias was not evaluated.

## Results

### Baseline features

**Fig. 1:**
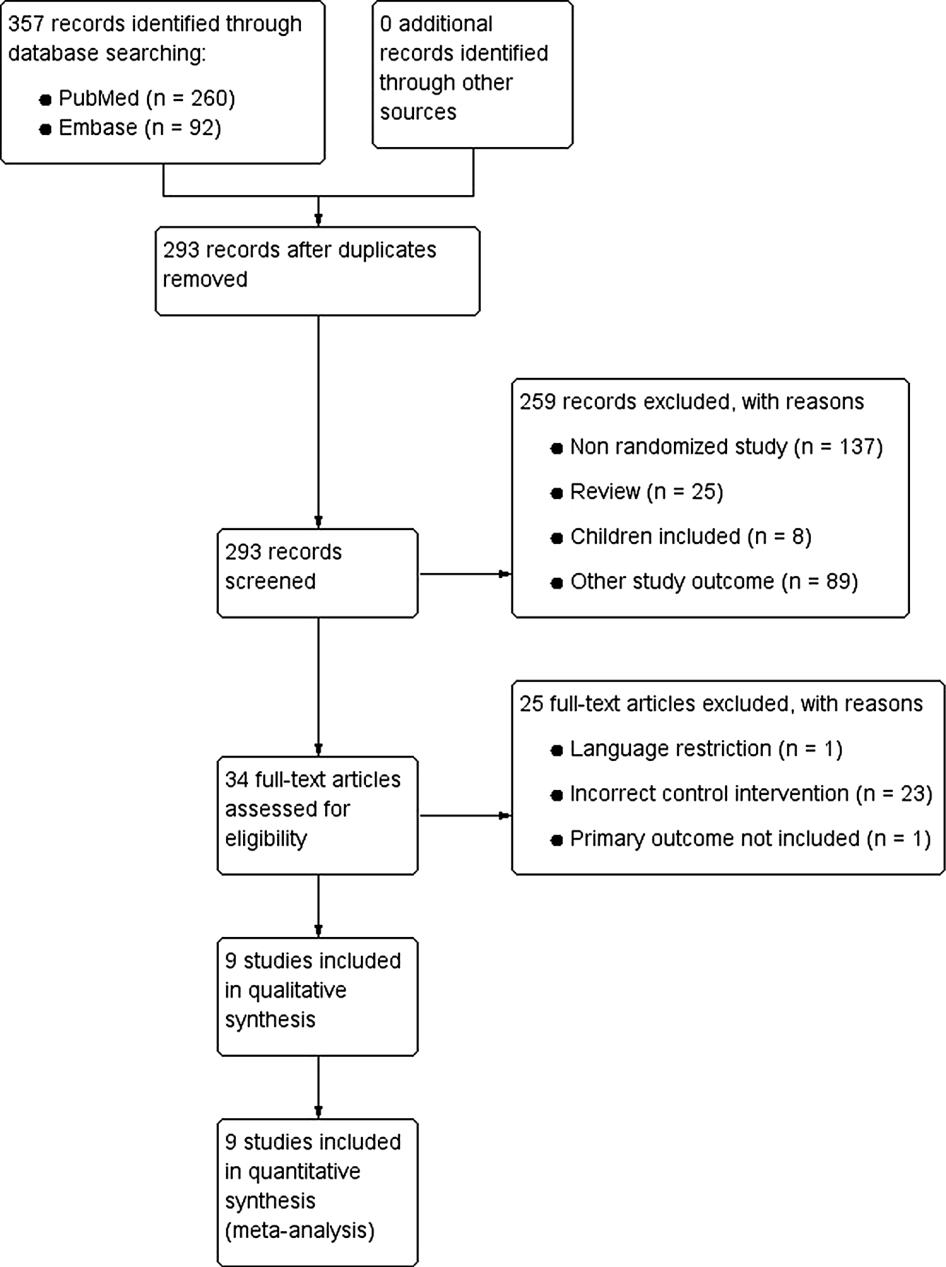
Study flow diagram

A total of 357 records were identified by the search of PubMed (*n* = 260) and EMBASE (*n* = 92). Figure 1 shows the article selection. After manual removal of duplicates, 293 records were screened. Of these, 259 records were discarded based on title and abstract because these papers investigated other study outcomes (*n* = 89), were non-randomized studies (*n* = 137), included children (*n* = 8) or were reviews (*n* = 25). For the remaining 34 records, full-text articles were retrieved and examined in detail for eligibility. Twenty-five additional studies were discarded, because one study could not be feasibly translated into English or Dutch, one study did not include our primary outcome measures, and twenty-three studies used an incorrect control group.

A total of nine RCTs published in English met the eligibility criteria and were included in the systematic review and meta-analysis [15–23]. The follow-up period of these studies ranged from 1 to 16 months, and the number of included patients ranged from 34 to 250. A total of 860 participants were randomized to SILC (*n* = 430) or conventional LC (*n* = 430). Main inclusion criteria comprised adults (18 years or older) with an ASA grade between I and III, scheduled for elective cholecystectomy. Main exclusion criteria were pregnancy or lactation, coagulopathic patients, acute cholecystitis or previous upper abdominal surgery. Countries of origin were Switzerland, Turkey, Denmark, Egypt, Brazil, Germany and Italy. There were six unicentre and three multicentre studies. All studies compared SILC with conventional LC. Successful completion of SILC ranged between 72 and 100%. Detailed information on the characteristics of included studies is presented in Table 1.

**Table 1:** Basic study and patient characteristics

Reference	Sample size		Age, mean, years		Sex male/female		BMI (kg/m^2^)		ASA grade (I:II:III)		Successful completion SILC procedure (%)
	SILC	LC	SILC	LC	SILC	LC	SILC	LC	SILC	LC	
Lurje [20]	48	48	48	44	15:33	19:29	25	26	37:10:1	31:17:0	90
Sulu [23]	30	30	48	44	9:21	12:18	30.3	28.54	12:15:3	16:14:0	NA
Jørgensen [17]	60	60	46	46	0:60	0:60	26.6	24	43:16:1	36:24:0	72
Abd Ellatif [15]	125	125	48	47	30:95	37:88	26.9	29.5	75:35:15	71:34:20	97
Luna [19]	20	20	NA	NA	NA	NA	NA	NA	NA	NA	90
Saad [21]	35	35	45	49	28:7	26:9	25.4	25.4	14:20:1	13:21:1	97
Sinan [22]	17	17	48	48	4:13	8:9	27.3	27.2	NA	NA	100
Bucher [16]	75	75	42	44	NA	NA	26	25	NA	NA	97
Lirici [18]	20	20	45	50	6:14	6:14	25	27	5:14:1	4:12:4	90

*ASA grade* American Society of Anaesthesiologists grade; *BMI* body mass index; *LC* laparoscopic cholecystectomy; *NA* not available; *SILC* single-incision laparoscopic cholecystectomy

* Age and sex ratio are presented as SILC/LC

### Quality assessment of the included studies

**Fig. 2:**
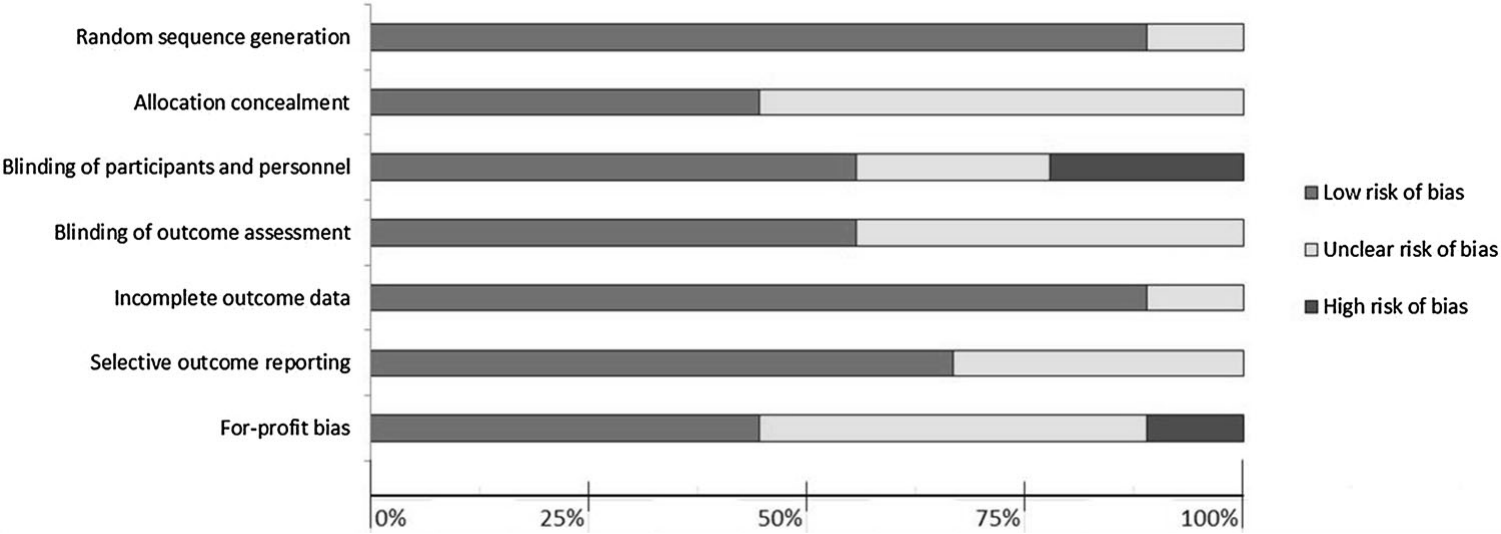
Risk of bias summary

**Fig. 3:**
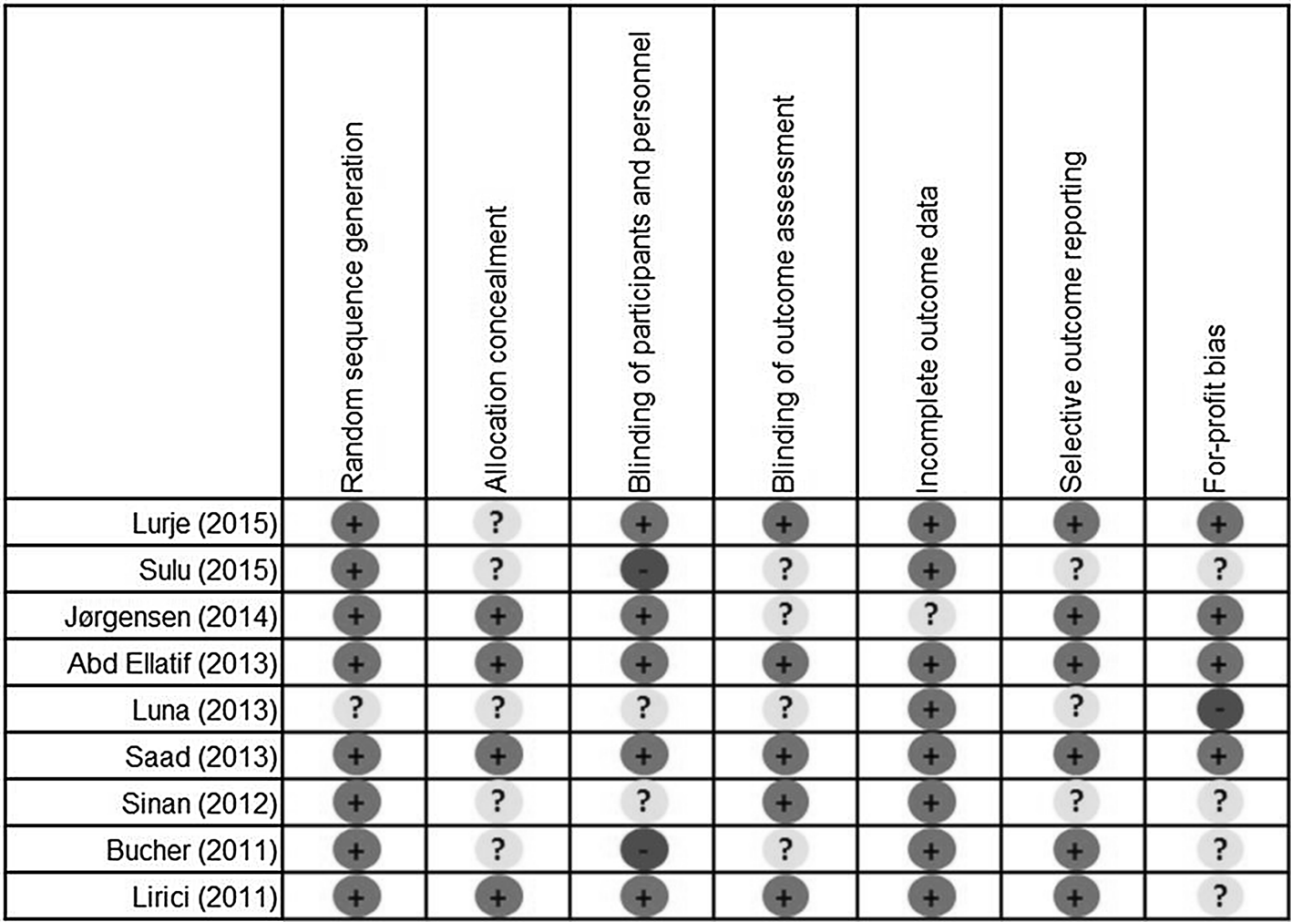
Risk of bias in individual studies

The risk of bias per domain and in individual studies is summarized in Figs. 2 and 3. A considerable heterogeneity between studies was observed. Overall, multiple studies were at “high risk” or “unclear risk” of bias. The only studies with “low risk” of bias were the studies conducted by Abd Ellatif et al. [15] and Saad et al. [21].

### Meta-analysis

#### Primary outcome measures

##### Mortality and morbidity

**Fig. 4:**
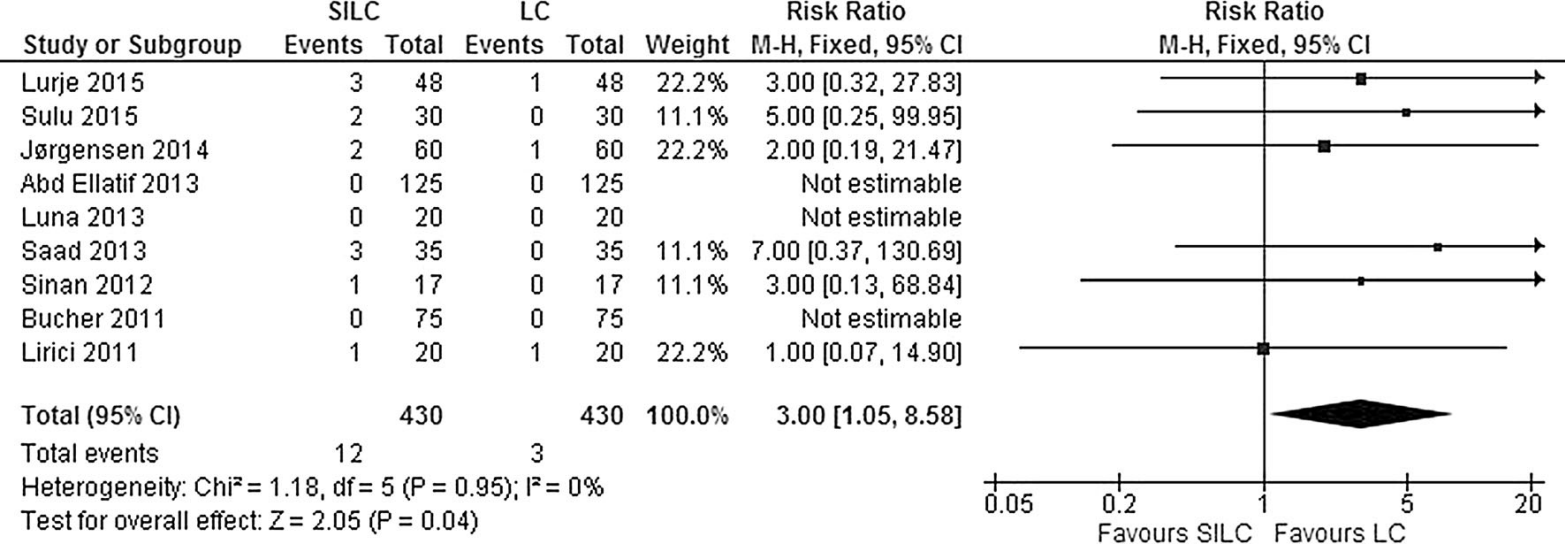
Forrest plot of serious adverse events for single-incision laparoscopic cholecystectomy (SILC) versus conventional laparoscopic cholecystectomy (LC). A Mantel–Haenszel fixed-effect model was used to calculate risk ratios with 95% confidence intervals

**Fig. 5:**
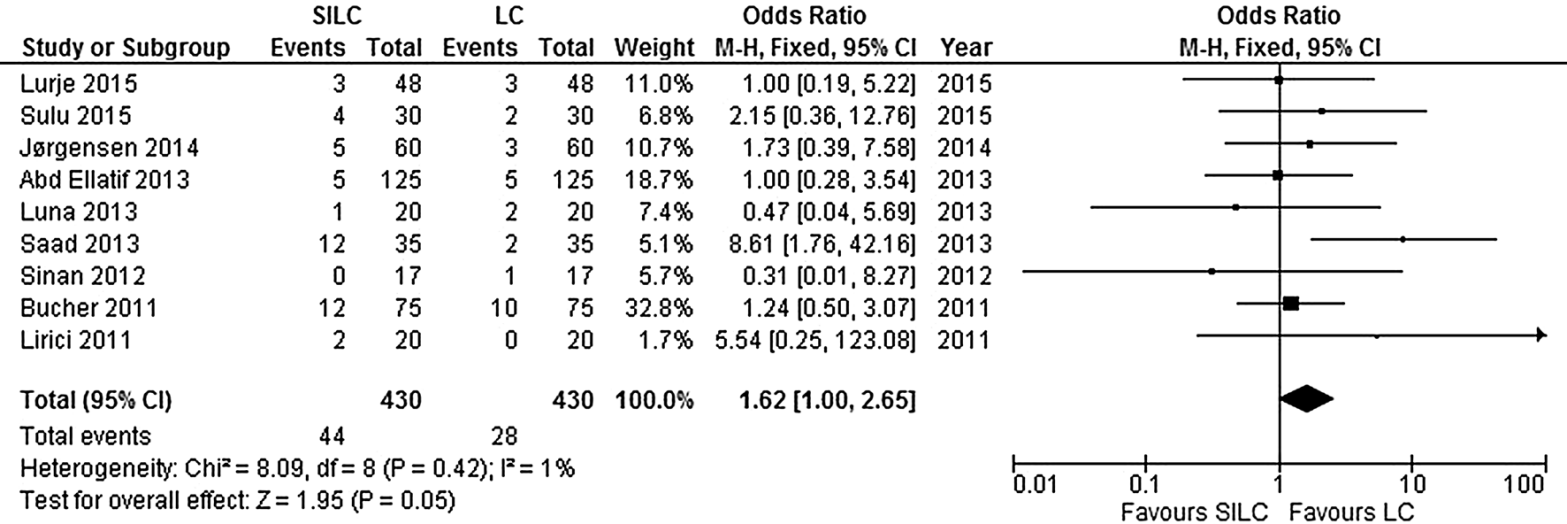
Forrest plot of mild adverse events for single-incision laparoscopic cholecystectomy (SILC) versus conventional laparoscopic cholecystectomy (LC). A Mantel–Haenszel fixed-effect model was used to calculate risk ratios with 95% confidence intervals

The data were available from all nine studies. No mortality was reported in either SILC or conventional LC. Serious adverse events included bile duct injury, re-operations, intra-abdominal collections or bile leaks requiring drainage or infected intra-abdominal collections. Mild adverse events include complications such as wound infections, bile leaks or abdominal collections that were easily treated or settled spontaneously. The pooled risk ratio shows a significant difference in the occurrence of serious adverse events, with more events occurring in the SILC group (RR 3.00; 95% CI 1.05–8.58) (Fig. 4). There was no significant heterogeneity between studies for this outcome (*χ* ^2^ = 1.18, *df* = 5, *I* ^2^ = 0%). In addition, more mild adverse events occurred in the SILC group (RR 1.55; 95% CI 0.99–2.42) (Fig. 5). There was no significant heterogeneity between studies (*χ* ^2^ = 7.25, *df* = 8, *I* ^2^ = 0%). Table 2 presents an overview of bile duct/vessel injury and port-site hernia after SILC and LC.

**Table 2:** Bile duct/vessel injury and port-site hernia after SILC and LC

Reference Year	Sample size	Biliary duct/vessel injury		Port-site hernia		Follow-up (months)	Method of SILC
		SILC	LC	SILC	LC		
Lurje [20] 2015	98	0	0	2	0	12	Transumbilical incision of 20–25 mm for SILS™ PT12 Port (Covidien Inc., Norwalk, California, USA) with 4 openings
Sulu [23] 2015	60	NA	NA	2	0	18	Transumbilical incision for SILS^®^ port (Covidien, Mansfield MA)
Jørgensen [17] 2014	120	1	0	1	1	12	Transumbilical incision of 25–30 mm for SILS port^®^ (Covidien, Mansfield, Massachusetts, USA) with 3 openings
Abd Ellatif [15] 2013	250	0	0	0	0	6	Two transumbilical ports, one of 10 mm and one of 5 mm
Luna [19] 2013	40	0	0	0	0	1	Transumbilical incision for the SITRACC^®^ device (EDLO, Rio Grande do Sul, Brazil) with one 10-mm and three 5-mm ports
Saad [21] 2013	70	0	0	1	0	12	Intra-umbilical 20-mm incision for SILS port^®^ (Covidien, Norwalk, Connecticut, USA) with 3 openings
Sinan [22] 2012	34	0	0	1	0	6	Intra-umbilical 25-mm incision for SILS port^®^ (Covidien, Norwalk, Connecticut, USA) with 3 openings
Bucher [16] 2011	150	0	0	0	0	1	Intra-umbilical 15-mm incision for TriPort^®^ (Advanced Surgical Concepts, Wicklow, Ireland) with 3 openings
Lirici [18] 2011	40	0	1	NA	NA	1	Intra-umbilical 20-mm incision for TriPort^®^ (Olympus America, Center Valley, PA) with 3 openings

*LC* laparoscopic cholecystectomy; *N*: *NA*: not available; *SILC* single-incision laparoscopic cholecystectomy

#### Secondary patient-reported outcome measures

**Fig. 6:**
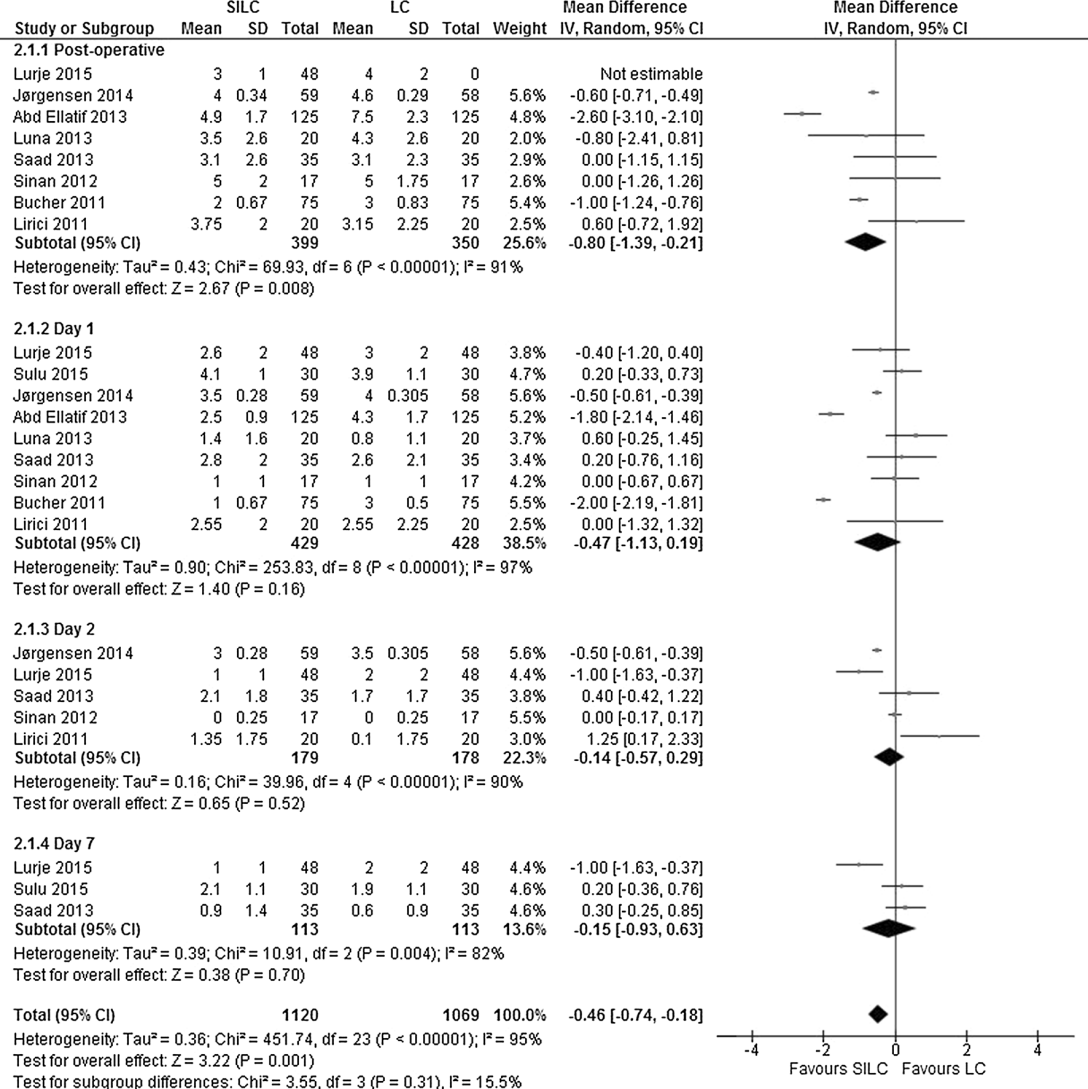
Forrest plot of post-operative pain for single-incision laparoscopic cholecystectomy (SILC) versus conventional laparoscopic cholecystectomy (LC). An inverse variance random-effects model was used to calculate mean difference with 95% confidence intervals

*VAS pain score*: All nine studies investigated post-operative pain with an 11-point VAS scale (ranging from 0 to 10, with a high score indicating more pain) at different time points (postoperatively and at days 1, 2 and 7 after surgery). Overall, there was a significant lower pain score in favour of SILC (MD −0.46; 95% CI −0.74 to −0.18). There was a considerable heterogeneity between the studies (*χ* ^2^ = 451.74, *df* = 23, *I* ^2^ = 95%). The calculated overall effect was due to a significant difference in pain postoperatively. At days 1, 2 and 7, no difference was found (Fig. 6).

**Fig. 7:**
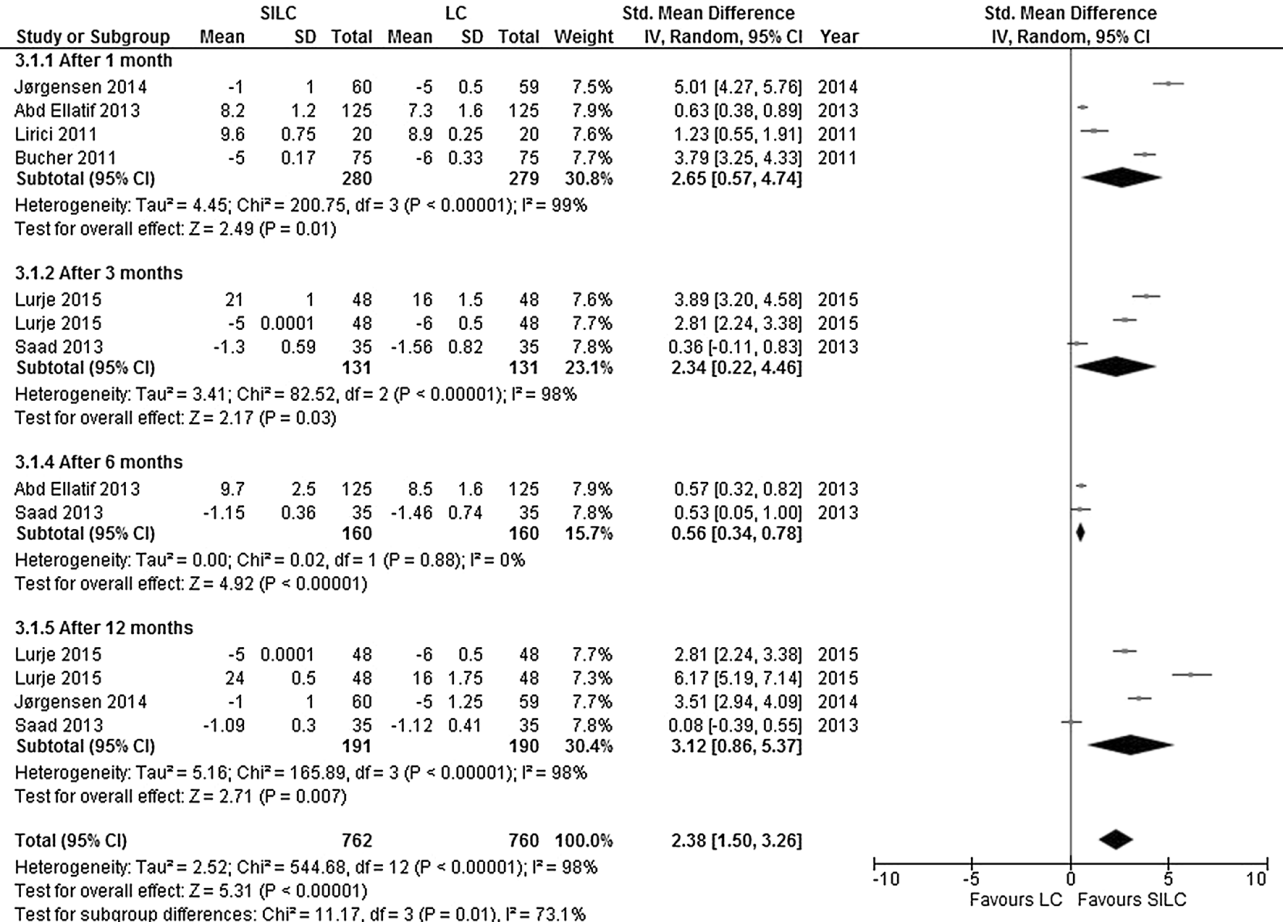
Forrest plot of cosmesis for single-incision laparoscopic cholecystectomy (SILC) versus conventional laparoscopic cholecystectomy (LC). An inverse variance random-effects model was used to calculate standardized mean difference with 95% confidence intervals

*Cosmesis*: Six studies explored the cosmetic outcome at different time points (after 1, 3, 6 or 12 months). Cosmesis was assessed with scales using a high or low score for better cosmetic results. Scales with a high score meaning a better result included a VAS scale (ranging from 0 to 10) [15, 18] and a Cosmesis Score (ranging from 3 to 24) [20]. Scales with a low score meaning a better result included a Body Image Scale (ranging from 5 to 20) [16, 20], a Numerical Rating Scale (ranging from 0 to 10) [17] and a Cosmesis Score (ranging from 1 to 5) [21]. Cosmesis was significantly better in the SILC group at all time points, and the overall SMD was in favour of SILC (SMD 2.38; 95% CI 1.50–3.26). The heterogeneity between studies was considerable (*χ* ^2^ = 544.68, *df* = 12, *I* ^2^ = 98%) (Fig. 7).

**Fig. 8:**
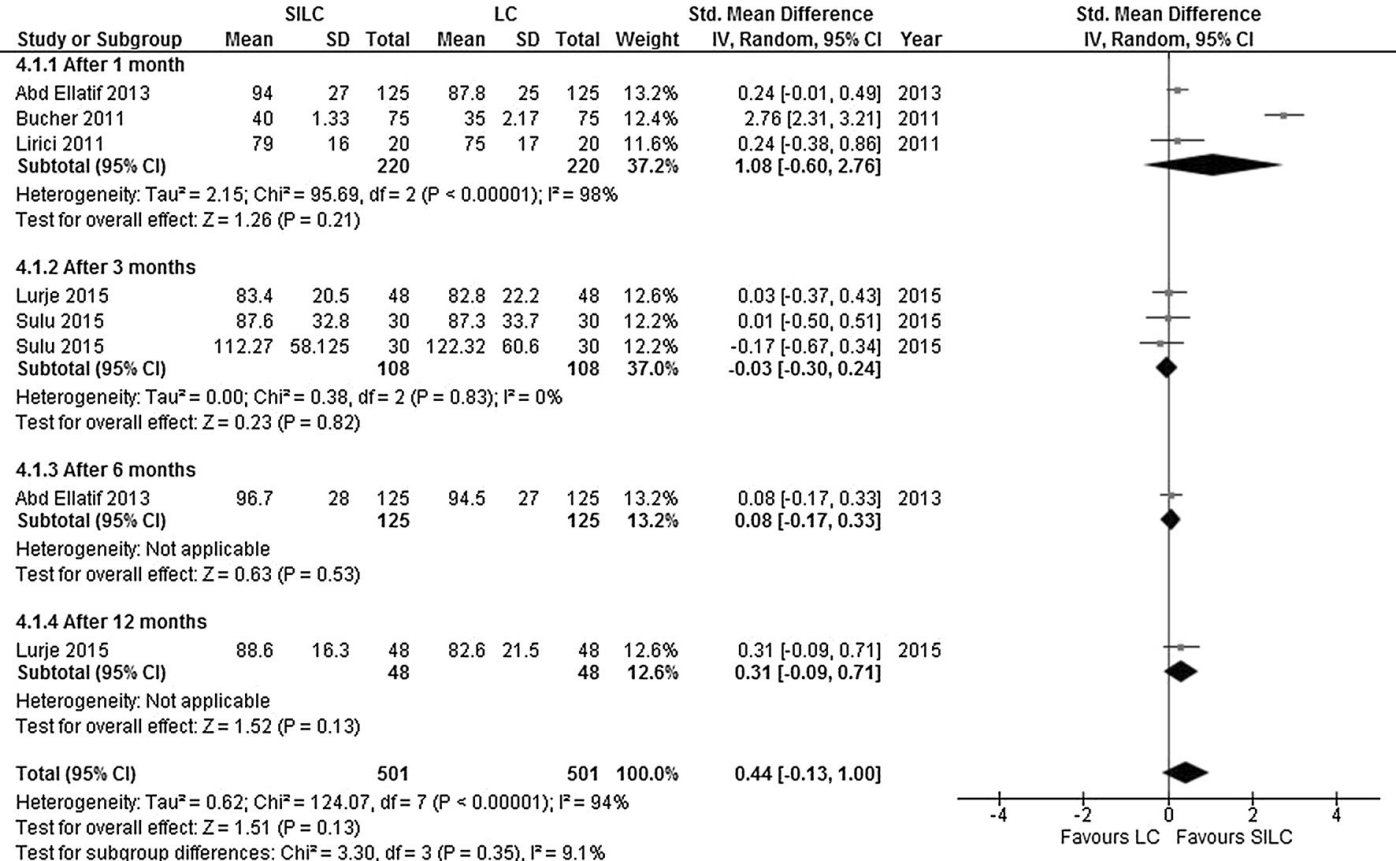
Forrest plot of quality of life for single-incision laparoscopic cholecystectomy (SILC) versus conventional laparoscopic cholecystectomy (LC). An inverse variance random-effects model was used to calculate standardized mean difference with 95% confidence intervals

*Quality of life*: Five studies explored quality of life at different time points (after 1, 3, 6 or 12 months). The questionnaires used for quality of life assessment included EQ-5D [15], GIQLI [23], SF-12 [16] and SF-36 [18, 20, 23]. There was no significant difference in quality of life (SMD 0.44; 95% CI −0.13 to 1.00). The heterogeneity between studies was high (*χ* ^2^ = 124.07, *df* = 7, *I* ^2^ = 94%) (Fig. 8).

#### Secondary procedure-related outcome measures

##### Conversion to open cholecystectomy:

**Fig. 9:**
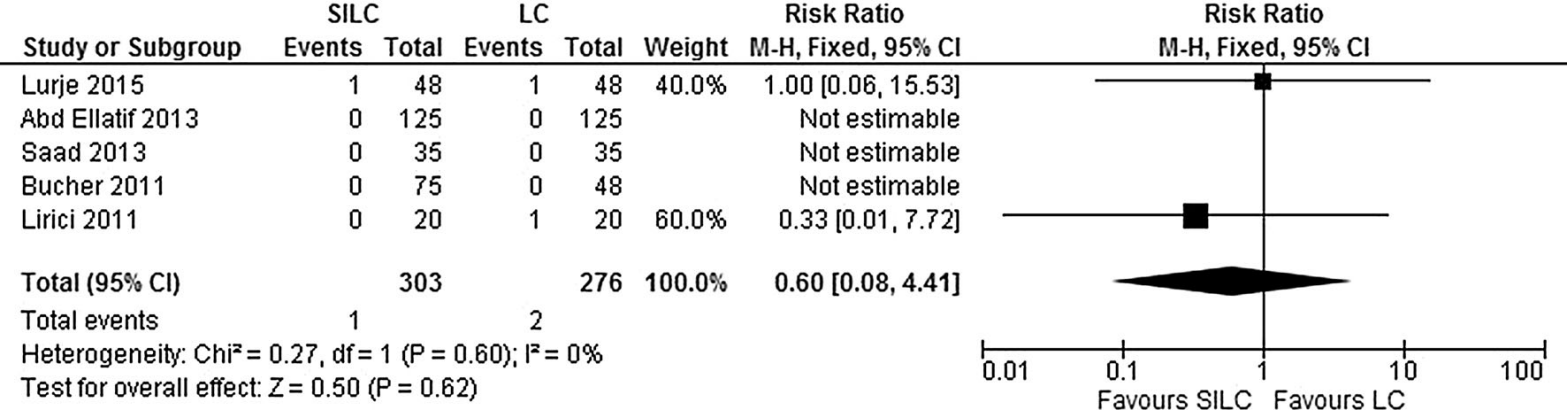
Forrest plot of conversion to open cholecystectomy for single-incision laparoscopic cholecystectomy (SILC) versus conventional laparoscopic cholecystectomy (LC). A Mantel–Haenszel fixed-effect model was used to calculate risk ratios with 95% confidence intervals

Conversion to open cholecystectomy was addressed in five studies. There was no significant difference in the proportion of conversion between both groups (RR 0.62; 95% CI 0.08–4.91). No significant heterogeneity between studies was found (*χ* ^2^ = 0.27, *df* = 1, *I* ^2^ = 0%) (Fig. 9).

**Fig. 10:**
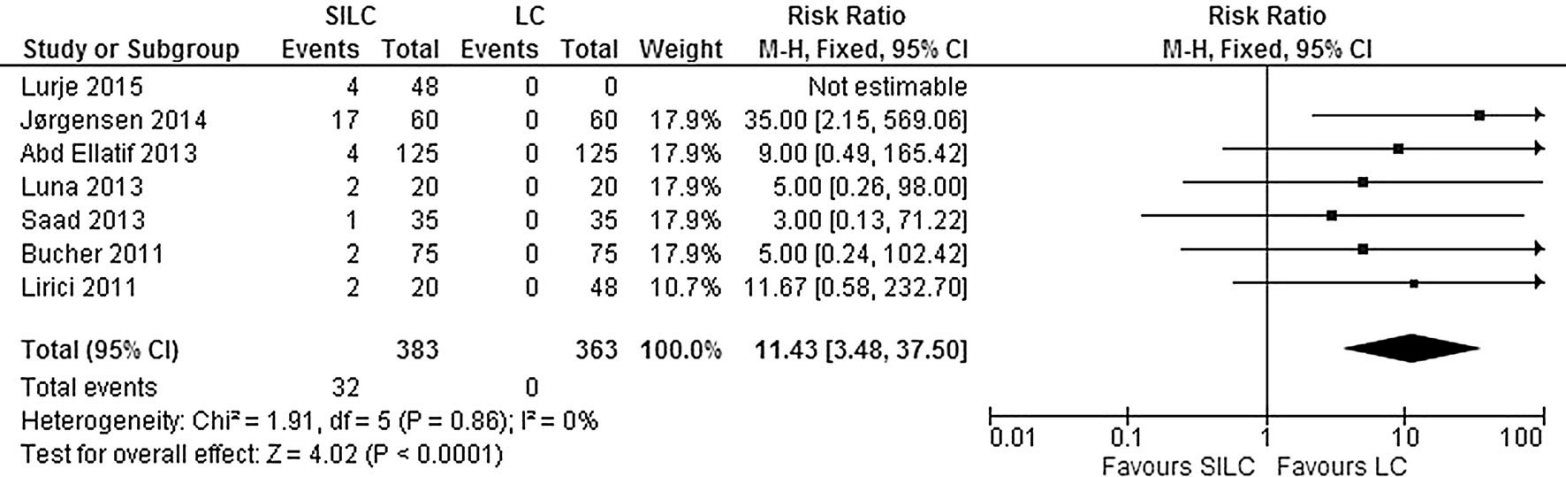
Forrest plot of additional ports needed for single-incision laparoscopic cholecystectomy (SILC) versus conventional laparoscopic cholecystectomy (LC). A Mantel–Haenszel fixed-effect model was used to calculate risk ratios with 95% confidence intervals

*Additional ports needed*: Seven studies explored the need of additional ports in both groups during surgery. The need for additional ports was significantly higher in the SILC group (RR 11.43; 95% CI 3.48–37.50). There was no significant heterogeneity between studies (*χ* ^2^ = 1.91, *df* = 5, *I* ^2^ = 0%) (Fig. 10).

**Fig. 11:**
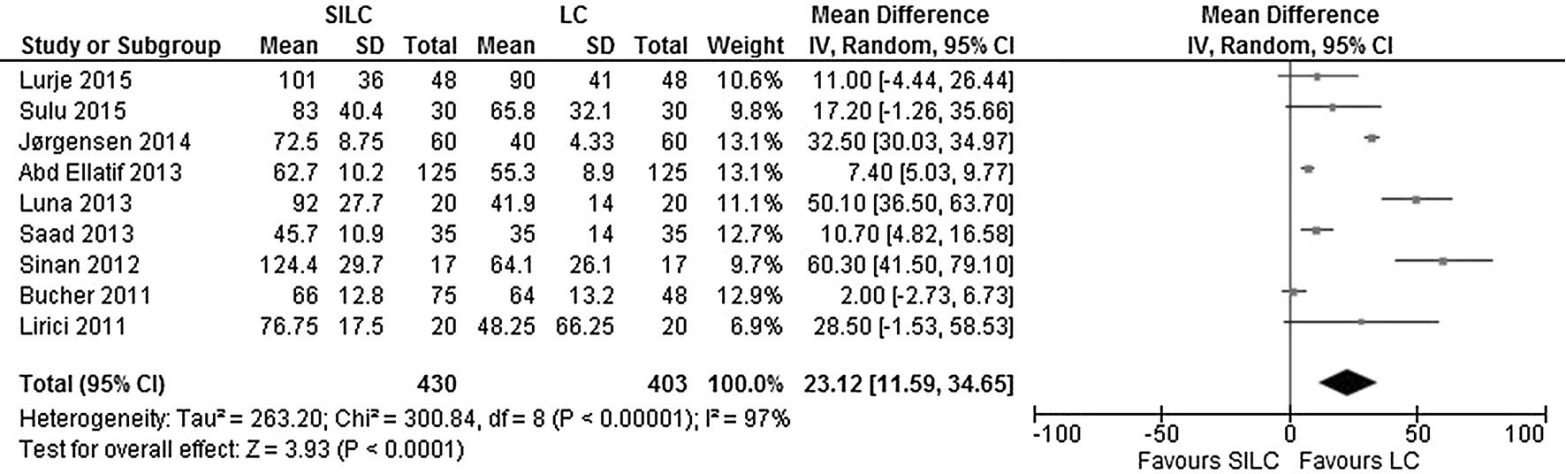
Forrest plot of operating time in minutes for single-incision laparoscopic cholecystectomy (SILC) versus conventional laparoscopic cholecystectomy (LC). An inverse variance random-effects model was used to calculate mean difference with 95% confidence intervals

*Operating time*: All nine studies reported the operating time. According to the calculated mean difference, there was a significant longer operating time in the SILC group (MD 23.12; 95% CI 11.59–34.65). The heterogeneity between studies was considerable (*χ* ^2^ = 300.84, *df* = 8, *I* ^2^ = 97%) (Fig. 11).

**Fig. 12:**
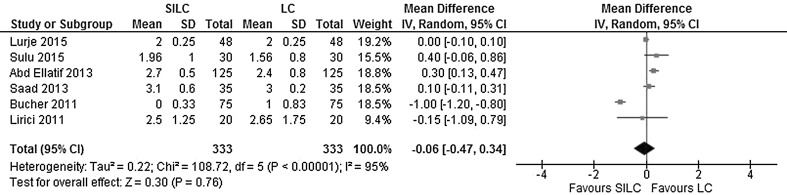
Forrest plot of hospital stay longer than 1 day for single-incision laparoscopic cholecystectomy (SILC) versus conventional laparoscopic cholecystectomy (LC). An inverse variance random-effects model was used to calculate mean difference with 95% confidence intervals

*Hospital stay*: A hospital stay longer than 24 h was reported in six studies. There was no significant difference observed between the groups (MD −0.06; 95% CI −0.47 - 0.34). The heterogeneity between studies was considerable (*χ* ^2^ = 108.72, *df* = 5, *I* ^2^ = 95%) (Fig. 12).

## Discussion

This systematic review included and analysed nine RCTs with total of 860 patients in order to assess safety, patient-reported outcome measures and feasibility of SILC versus conventional LC in patients undergoing elective cholecystectomy for gallstone-related disease.

The results show advantages of the SILC procedure above the LC procedure regarding post-operative pain and cosmesis. At the same time, more adverse events and shortcomings in feasibility occurred in the SILC group.

Less post-operative pain and improved cosmesis in favour of the SILC procedure did not have any impact on quality of life as no differences were observed for this outcome between the two groups. Post-operative pain was significantly lower, and the overall pain score was in favour of SILC. However, at days 1, 2 and 7, no difference was found, suggesting that the decrease in post-operative pain is mainly important in the very early post-operative period. Cosmesis was significantly better in the SILC group at all time points. There were no significant differences in the conversion to open cholecystectomy or hospital stay longer than 24 h. Operating time and the need for additional ports were significantly higher in the SILC group. Also significantly more serious and more mild adverse events occurred in the SILC group, indicating that SILC might not be as safe as the conventional LC. No mortality was found in both groups. However, it should be noted that most RCTs included only patients with a low ASA grade who were scheduled for elective cholecystectomy, thereby reducing the risk of mortality.

These results indicate that SILC offers benefits in terms of decreased post-operative pain and improved cosmesis, but it does not seem to improve quality of life or decrease the length of hospital stay. In contrast, SILC appears to increase the risk of adverse events, is frequently in need of additional ports and prolongs the operation time.

The presented study results must be interpreted with caution. Overall, the evidence from these nine RCTs is not strong enough to provide reliable results because multiple studies were at high risk or uncertain risk of bias, and a considerable heterogeneity between studies was observed. The only studies with low risk of bias were the studies conducted by Abd Ellatif et al. [15] and Saad et al. [21]. Moreover, the sample size was low in most studies.

Another limitation is due to the fact that the definition of cosmesis and quality of life were different across the studies. In addition, the learning curve associated with the adoption to SILC could induce differences in the operation time and the need for additional ports, because the experience of surgeons differed across studies.

There are some serious methodological issues, which might compromise the validity of the outcomes. Blinding of patients and personnel was at high risk of bias in most studies; objective patient-reported outcome measures such as post-operative pain, cosmesis and quality of life are susceptible for performance bias. In addition, multiple studies performed SILC with a specific device made by a company. Since for-profit bias was poorly evaluated in these studies, reliability of the results can be questionable due to possible conflict of interest. Moreover, the follow-up period was relatively short in most studies. In order to adequately assess long-term safety and cosmesis, a follow-up period of at least 1 year is desirable.

It should be also noted that a very specific group of patients was studied. Most RCTs only included patients scheduled for elective cholecystectomy with a low ASA grade, thereby disregarding emergency cholecystectomy. Moreover, most studies excluded obese patients. Therefore, the results presented in this systematic review are only applicable in non-obese patients with low ASA grade scheduled for elective cholecystectomy. There was no consensus regarding the technique and the devices used for SILC. This might have introduced inconsistencies in the results of this review.

Due to a relatively short follow-up in the included studies, the problem of trocar-site hernia might be underestimated, as this might occur even years after the surgery. In SILC technique, the size of the incision is bigger than in multiport approach so one could expect more incisional hernias. This could have a negative impact on the cosmetic outcome after SILC. Also in obese patients, one could expect a higher risk of hernias. These patients were, however, not included in the studies.

Limitation regarding the design of this systematic review might be the inclusion of studies published in English only. Further, in this review conventional four-port LC is defined as two 10-mm and two 5-mm ports. Currently, surgeons also use three 5-mm ports and one 10-mm port or even three ports only, instead of four. Inclusion of studies using these techniques could alter some of the conclusions of this study.

In the literature, several reviews exist on SILC versus conventional LC in patients scheduled for cholecystectomy for gallstone-related disease, but only one review performed by Gurusamy et al. [3] used the same definition of conventional LC as was used in this study. The above-mentioned review suggested that there was no significant difference in the proportion of mortality, serious complications, quality of life, cosmesis and conversion to open cholecystectomy or length of hospital stay. In addition, the authors reported a significant longer operation duration [3]. Some of the other published reviews suggest that SILC is safe and effective [4, 6], while others do not see any advantages or empathize to be cautious with SILC [5, 8]. Results on post-operative pain were controversial [4, 6, 8].

In conclusion, low-quality evidence indicates that safety of SILC is debatable and that this procedure does not provide any clear advantages over conventional LC, except for a decrease in post-operative pain and an improvement in cosmetic result. However, we could not establish the impact of these outcomes on the quality of life. Therefore, it seems questionable whether the mentioned advantages of the SILC procedure outweigh the disadvantages such as occurrence of adverse events, the prolonged operating time and the frequent need for additional ports.

Well-designed RCTs with high-quality evidence and a follow-up period longer than 1 year are necessary to establish the safety profile and clinical benefits of SILC. These studies should also broaden the applicability of SILC by including patients with obesity and cases requiring emergency cholecystectomy. Furthermore, cost-effectiveness should be addressed in an economic evaluation, since high-quality data on economic aspects are very sparse.
